# *N*-Benzo­ylbenzene­sulfonamide

**DOI:** 10.1107/S1600536809037222

**Published:** 2009-09-26

**Authors:** B. Thimme Gowda, Sabine Foro, P. A. Suchetan, Hartmut Fuess

**Affiliations:** a Department of Chemistry, Mangalore University, Mangalagangotri 574 199, Mangalore, India; b Institute of Materials Science, Darmstadt University of Technology, Petersenstrasse 23, D-64287, Darmstadt, Germany

## Abstract

In the crystal structure of the title compound, C_13_H_11_NO_3_S, the conformation of the N—H bond in the C—SO_2_—NH—C(O)—C segment is *anti* to the C=O bond. The molecule is twisted at theN atom with a dihedral angle of 86.5(1)° between the sulfonyl benzene ring and the —SO_2_—NH—C=O segment. Furthermore, the dihedral angle between the two benzene rings is 80.3(1)°. The crystal structure features inversion-related dimers linked by pairs of N—H⋯O(S) hydrogen bonds.

## Related literature

For related structures, see: Gowda *et al.* (2008*a* ▶,*b* ▶; 2009 ▶).
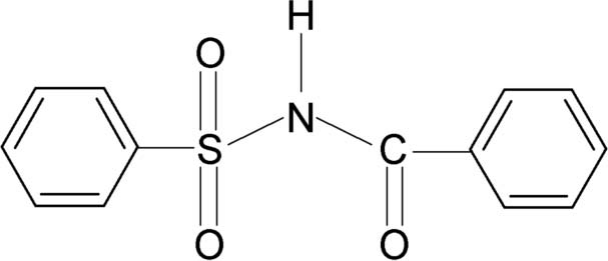


## Experimental

### 

#### Crystal data

C_13_H_11_NO_3_S
*M* *_r_* = 261.29
Triclinic, 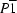

*a* = 5.8396 (7) Å
*b* = 10.178 (1) Å
*c* = 10.405 (1) Å
α = 90.187 (8)°
β = 99.074 (9)°
γ = 99.617 (9)°
*V* = 601.83 (11) Å^3^
*Z* = 2
Cu *K*α radiation
μ = 2.40 mm^−1^
*T* = 299 K
0.50 × 0.33 × 0.05 mm

#### Data collection

Enraf–Nonius CAD-4 diffractometer
Absorption correction: ψ scan North *et al.*, 1968 ▶ *T*_min_ = 0.380, *T*_max_ = 0.889
2354 measured reflections
2125 independent reflections
1962 reflections with *I* > 2σ(*I*)
*R*_int_ = 0.011
3 standard reflections frequency: 120 min intensity decay: 1.0%

#### Refinement

*R*[*F* ^2^ > 2σ(*F* ^2^)] = 0.055
*wR*(*F* ^2^) = 0.171
*S* = 1.18
2125 reflections
167 parameters
7 restraints
H atoms treated by a mixture of independent and constrained refinement
Δρ_max_ = 0.65 e Å^−3^
Δρ_min_ = −0.36 e Å^−3^

### 

Data collection: *CAD-4-PC* (Enraf–Nonius, 1996 ▶); cell refinement: *CAD-4-PC*; data reduction: *REDU4* (Stoe & Cie, 1987 ▶); program(s) used to solve structure: *SHELXS97* (Sheldrick, 2008 ▶); program(s) used to refine structure: *SHELXL97* (Sheldrick, 2008 ▶); molecular graphics: *PLATON* (Spek, 2009 ▶); software used to prepare material for publication: *SHELXL97*.

## Supplementary Material

Crystal structure: contains datablocks I, global. DOI: 10.1107/S1600536809037222/tk2540sup1.cif

Structure factors: contains datablocks I. DOI: 10.1107/S1600536809037222/tk2540Isup2.hkl

Additional supplementary materials:  crystallographic information; 3D view; checkCIF report

## Figures and Tables

**Table 1:** Hydrogen-bond geometry (Å, °)

*D*—H⋯*A*	*D*—H	H⋯*A*	*D*⋯*A*	*D*—H⋯*A*
N1—H1*N*⋯O1^i^	0.79 (3)	2.22 (3)	2.981 (4)	163 (4)

Symmetry code: (i) 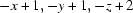.

**Fig. 1.**
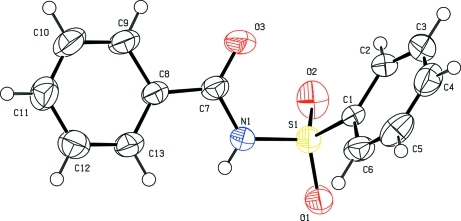
Molecular structure of (I), showing the atom labelling scheme and displacement ellipsoids are drawn at the 50% probability level.

**Fig. 2.**
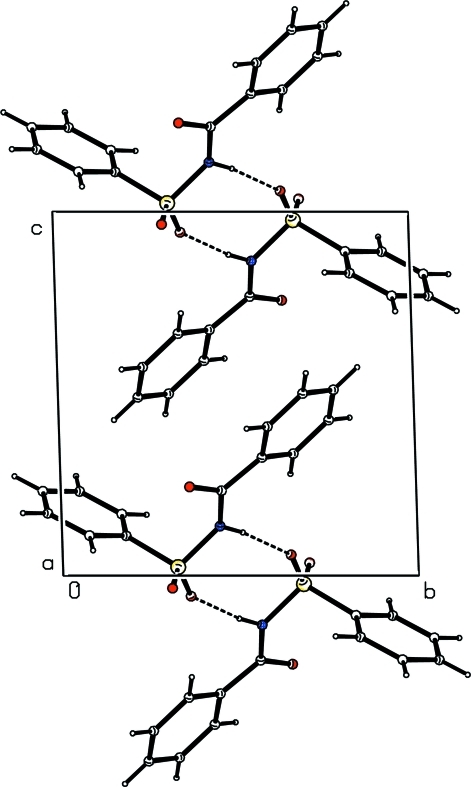
Molecular packing of (I) with hydrogen bonding shown as dashed lines.

## supplementary crystallographic information

### Comment

Diaryl acylsulfonamides are known as potent anti-tumor agents against a broad spectrum of human tumor xenografts (colon, lung, breast, ovary, and prostate) in nude mice. As part of a study of the effect of ring and the side chain substituents on the solid-state structures of *N*-aromatic sulfonamides (Gowda *et al.*, 2008*a*,*b*; 2009), in the present work the structure of *N*-(benzoyl)benzenesulfonamide (I) has been determined (Fig. 1). The conformation of the N—H bond in the structure is *anti* to the C═O bond in the side-chain, similar to that observed in the acid anilides. The conformation of the N—C bond in the C—SO_2_—NH—C(O) segment of the structure has "*gauche*" torsions with respect to the SO bonds (Fig. 1). The molecule is twisted at the C(O) atom with the C—SO_2_—NH—C(O) torsion angle being -66.9 (3)°. The packing of molecules *via* N—H···O(S) hydrogen bonds (Table 1) into supramolecular dimers is shown in Fig. 2.

### Experimental

Compound (I) was prepared by refluxing a mixture of benzoic acid, benzene sulfonamide and phosphorous oxy chloride for 5 h on a water bath. The resultant mixture was cooled and poured into ice-cold water. The solid obtained was filtered and washed thoroughly with water and then dissolved in sodium bicarbonate solution. Compound (I) was later reprecipitated by acidifying the filtered solution with dilute HCl. The filtered and dried solid was recrystallized to the constant melting point. The compound was characterized by its characteristic aromatic C—H stretching (3061.1 cm^-1^), carbonyl C═O (1696.7 cm^-1^), N—H stretching (3280.1 cm^-1^), symmetric (1176.3 cm^-1^), and asymmetric SO_2_ (1335.1 cm^-1^) infrared absorption frequencies.

Long colorless plates of (I) were obtained from a slow evaporation of its toluene solution at room temperature.

### Refinement

The H atom of the NH group was located in a difference map and and later restained to N—H = 0.86 (4) Å. The other H atoms were positioned with idealized geometry using a riding model with C—H = 0.93 Å. All H atoms were refined with isotropic displacement parameters (set to 1.2 times of the *U*_eq_ of the parent atom).

The U_ij_ components of C5 were restrained to approximate isotropic behavoir.

### Crystal data

**Table:**

C_13_H_11_NO_3_S	*Z* = 2
*M**_r_* = 261.29	*F*(000) = 272
Triclinic, *P*1	*D*_x_ = 1.442 Mg m^−^^3^
Hall symbol: -P 1	Cu *K*α radiation, λ = 1.54180 Å
*a* = 5.8396 (7) Å	Cell parameters from 25 reflections
*b* = 10.178 (1) Å	θ = 4.3–22.9°
*c* = 10.405 (1) Å	µ = 2.40 mm^−^^1^
α = 90.187 (8)°	*T* = 299 K
β = 99.074 (9)°	Long plate, colorless
γ = 99.617 (9)°	0.50 × 0.33 × 0.05 mm
*V* = 601.83 (11) Å^3^	

### Data collection

**Table:**

Enraf–Nonius CAD-4 diffractometer	1962 reflections with *I* > 2σ(*I*)
Radiation source: fine-focus sealed tube	*R*_int_ = 0.011
graphite	θ_max_ = 66.9°, θ_min_ = 4.3°
ω/2θ scans	*h* = 0→6
Absorption correction: ψ scan North *et al.*, 1968	*k* = −12→11
*T*_min_ = 0.380, *T*_max_ = 0.889	*l* = −12→12
2354 measured reflections	3 standard reflections every 120 min
2125 independent reflections	intensity decay: 1.0%

### Refinement

**Table:**

Refinement on *F*^2^	Secondary atom site location: difference Fourier map
Least-squares matrix: full	Hydrogen site location: inferred from neighbouring sites
*R*[*F*^2^ > 2σ(*F*^2^)] = 0.055	H atoms treated by a mixture of independent and constrained refinement
*wR*(*F*^2^) = 0.171	*w* = 1/[σ^2^(*F*_o_^2^) + (0.0862*P*)^2^ + 0.5464*P*] where *P* = (*F*_o_^2^ + 2*F*_c_^2^)/3
*S* = 1.18	(Δ/σ)_max_ = 0.008
2125 reflections	Δρ_max_ = 0.65 e Å^−^^3^
167 parameters	Δρ_min_ = −0.36 e Å^−^^3^
7 restraints	Extinction correction: *SHELXL97* (Sheldrick, 2008), Fc^*^=kFc[1+0.001xFc^2^λ^3^/sin(2θ)]^-1/4^
Primary atom site location: structure-invariant direct methods	Extinction coefficient: 0.024 (3)

### Special details

**Table:**

Geometry. All e.s.d.'s (except the e.s.d. in the dihedral angle between two l.s. planes) are estimated using the full covariance matrix. The cell e.s.d.'s are taken into account individually in the estimation of e.s.d.'s in distances, angles and torsion angles; correlations between e.s.d.'s in cell parameters are only used when they are defined by crystal symmetry. An approximate (isotropic) treatment of cell e.s.d.'s is used for estimating e.s.d.'s involving l.s. planes.
Refinement. Refinement of *F*^2^ against ALL reflections. The weighted *R*-factor *wR* and goodness of fit *S* are based on *F*^2^, conventional *R*-factors *R* are based on *F*, with *F* set to zero for negative *F*^2^. The threshold expression of *F*^2^ > σ(*F*^2^) is used only for calculating *R*-factors(gt) *etc*. and is not relevant to the choice of reflections for refinement. *R*-factors based on *F*^2^ are statistically about twice as large as those based on *F*, and *R*- factors based on ALL data will be even larger.

### Fractional atomic coordinates and isotropic or equivalent isotropic displacement parameters (Å^2^)

**Table:**

	*x*	*y*	*z*	*U*_iso_*/*U*_eq_	
C1	0.3607 (5)	0.8198 (3)	0.8908 (3)	0.0337 (6)	
C2	0.2344 (5)	0.9229 (3)	0.8901 (3)	0.0407 (7)	
H2	0.1021	0.9143	0.9307	0.049*	
C3	0.3058 (7)	1.0380 (3)	0.8289 (4)	0.0539 (9)	
H3	0.2219	1.1081	0.8283	0.065*	
C4	0.5004 (7)	1.0503 (4)	0.7686 (4)	0.0589 (10)	
H4	0.5482	1.1288	0.7274	0.071*	
C5	0.6248 (6)	0.9472 (4)	0.7688 (4)	0.0583 (10)	
H5	0.7553	0.9559	0.7266	0.070*	
C6	0.5584 (5)	0.8304 (4)	0.8311 (3)	0.0470 (8)	
H6	0.6442	0.7611	0.8329	0.056*	
C7	0.0114 (5)	0.5480 (3)	0.7670 (3)	0.0400 (7)	
C8	−0.0390 (5)	0.4316 (3)	0.6753 (3)	0.0368 (7)	
C9	−0.2525 (6)	0.4135 (4)	0.5908 (3)	0.0500 (8)	
H9	−0.3577	0.4717	0.5965	0.060*	
C10	−0.3098 (6)	0.3115 (4)	0.4997 (4)	0.0602 (10)	
H10	−0.4533	0.3009	0.4441	0.072*	
C11	−0.1570 (7)	0.2248 (4)	0.4897 (4)	0.0564 (9)	
H11	−0.1958	0.1557	0.4273	0.068*	
C12	0.0539 (7)	0.2408 (4)	0.5727 (4)	0.0589 (10)	
H12	0.1576	0.1818	0.5664	0.071*	
C13	0.1137 (6)	0.3429 (4)	0.6649 (3)	0.0492 (8)	
H13	0.2571	0.3526	0.7205	0.059*	
N1	0.2045 (5)	0.5551 (3)	0.8642 (3)	0.0436 (7)	
H1N	0.262 (7)	0.491 (3)	0.882 (4)	0.052*	
O1	0.4799 (5)	0.6424 (2)	1.0568 (3)	0.0629 (8)	
O2	0.0786 (5)	0.6940 (3)	1.0349 (3)	0.0597 (7)	
O3	−0.1033 (4)	0.6374 (3)	0.7564 (3)	0.0562 (7)	
S1	0.27661 (14)	0.67612 (7)	0.97654 (7)	0.0416 (3)	

### Atomic displacement parameters (Å^2^)

**Table:**

	*U*^11^	*U*^22^	*U*^33^	*U*^12^	*U*^13^	*U*^23^
C1	0.0246 (13)	0.0344 (14)	0.0396 (15)	0.0060 (10)	−0.0030 (11)	−0.0037 (11)
C2	0.0305 (15)	0.0399 (16)	0.0508 (18)	0.0101 (12)	−0.0009 (12)	−0.0029 (13)
C3	0.054 (2)	0.0397 (17)	0.064 (2)	0.0116 (15)	−0.0085 (17)	0.0005 (15)
C4	0.057 (2)	0.049 (2)	0.058 (2)	−0.0084 (16)	−0.0088 (18)	0.0092 (16)
C5	0.0344 (17)	0.081 (3)	0.054 (2)	−0.0073 (17)	0.0082 (15)	−0.0003 (18)
C6	0.0320 (16)	0.059 (2)	0.0508 (18)	0.0136 (14)	0.0041 (13)	−0.0049 (15)
C7	0.0280 (15)	0.0441 (17)	0.0469 (17)	0.0057 (12)	0.0030 (12)	0.0036 (13)
C8	0.0280 (14)	0.0415 (16)	0.0392 (15)	0.0050 (11)	0.0013 (11)	0.0046 (12)
C9	0.0314 (16)	0.063 (2)	0.0534 (19)	0.0125 (14)	−0.0044 (14)	−0.0042 (16)
C10	0.0408 (19)	0.076 (3)	0.056 (2)	0.0057 (17)	−0.0116 (16)	−0.0082 (18)
C11	0.061 (2)	0.056 (2)	0.0469 (19)	0.0034 (17)	−0.0017 (16)	−0.0092 (16)
C12	0.061 (2)	0.062 (2)	0.055 (2)	0.0257 (18)	−0.0035 (17)	−0.0090 (17)
C13	0.0401 (17)	0.056 (2)	0.0486 (18)	0.0154 (14)	−0.0082 (14)	−0.0067 (15)
N1	0.0414 (15)	0.0331 (13)	0.0525 (16)	0.0104 (11)	−0.0086 (12)	0.0001 (12)
O1	0.0787 (18)	0.0431 (13)	0.0567 (15)	0.0186 (12)	−0.0293 (13)	−0.0018 (11)
O2	0.0647 (16)	0.0593 (15)	0.0575 (15)	0.0008 (12)	0.0278 (13)	−0.0007 (12)
O3	0.0414 (13)	0.0604 (15)	0.0686 (16)	0.0248 (11)	−0.0031 (11)	−0.0111 (12)
S1	0.0460 (5)	0.0348 (5)	0.0405 (5)	0.0062 (3)	−0.0023 (3)	0.0006 (3)

### Geometric parameters (Å, °)

**Table:**

C1—C2	1.379 (4)	C8—C13	1.385 (5)
C1—C6	1.384 (4)	C8—C9	1.392 (4)
C1—S1	1.756 (3)	C9—C10	1.366 (5)
C2—C3	1.370 (5)	C9—H9	0.9300
C2—H2	0.9300	C10—C11	1.370 (6)
C3—C4	1.370 (6)	C10—H10	0.9300
C3—H3	0.9300	C11—C12	1.373 (5)
C4—C5	1.373 (6)	C11—H11	0.9300
C4—H4	0.9300	C12—C13	1.375 (5)
C5—C6	1.383 (5)	C12—H12	0.9300
C5—H5	0.9300	C13—H13	0.9300
C6—H6	0.9300	N1—S1	1.650 (3)
C7—O3	1.212 (4)	N1—H1N	0.79 (3)
C7—N1	1.383 (4)	O1—S1	1.432 (2)
C7—C8	1.479 (4)	O2—S1	1.425 (3)
			
C2—C1—C6	121.3 (3)	C10—C9—C8	121.0 (3)
C2—C1—S1	119.0 (2)	C10—C9—H9	119.5
C6—C1—S1	119.6 (2)	C8—C9—H9	119.5
C3—C2—C1	119.4 (3)	C9—C10—C11	120.4 (3)
C3—C2—H2	120.3	C9—C10—H10	119.8
C1—C2—H2	120.3	C11—C10—H10	119.8
C4—C3—C2	120.3 (3)	C10—C11—C12	119.4 (3)
C4—C3—H3	119.9	C10—C11—H11	120.3
C2—C3—H3	119.9	C12—C11—H11	120.3
C3—C4—C5	120.2 (3)	C11—C12—C13	120.8 (3)
C3—C4—H4	119.9	C11—C12—H12	119.6
C5—C4—H4	119.9	C13—C12—H12	119.6
C4—C5—C6	120.8 (3)	C12—C13—C8	120.2 (3)
C4—C5—H5	119.6	C12—C13—H13	119.9
C6—C5—H5	119.6	C8—C13—H13	119.9
C1—C6—C5	118.1 (3)	C7—N1—S1	122.6 (2)
C1—C6—H6	121.0	C7—N1—H1N	120 (3)
C5—C6—H6	121.0	S1—N1—H1N	114 (3)
O3—C7—N1	120.1 (3)	O2—S1—O1	119.09 (18)
O3—C7—C8	122.6 (3)	O2—S1—N1	110.97 (15)
N1—C7—C8	117.2 (3)	O1—S1—N1	103.63 (14)
C13—C8—C9	118.2 (3)	O2—S1—C1	108.38 (15)
C13—C8—C7	124.8 (3)	O1—S1—C1	109.23 (15)
C9—C8—C7	117.0 (3)	N1—S1—C1	104.55 (14)
			
C6—C1—C2—C3	0.1 (5)	C10—C11—C12—C13	−0.3 (6)
S1—C1—C2—C3	176.8 (2)	C11—C12—C13—C8	0.0 (6)
C1—C2—C3—C4	0.2 (5)	C9—C8—C13—C12	0.3 (5)
C2—C3—C4—C5	0.1 (5)	C7—C8—C13—C12	−177.3 (3)
C3—C4—C5—C6	−0.9 (5)	O3—C7—N1—S1	5.1 (4)
C2—C1—C6—C5	−0.9 (5)	C8—C7—N1—S1	−177.7 (2)
S1—C1—C6—C5	−177.5 (2)	C7—N1—S1—O2	49.8 (3)
C4—C5—C6—C1	1.3 (5)	C7—N1—S1—O1	178.7 (3)
O3—C7—C8—C13	164.9 (3)	C7—N1—S1—C1	−66.9 (3)
N1—C7—C8—C13	−12.2 (5)	C2—C1—S1—O2	−0.3 (3)
O3—C7—C8—C9	−12.7 (5)	C6—C1—S1—O2	176.3 (2)
N1—C7—C8—C9	170.2 (3)	C2—C1—S1—O1	−131.5 (2)
C13—C8—C9—C10	−0.3 (5)	C6—C1—S1—O1	45.2 (3)
C7—C8—C9—C10	177.5 (3)	C2—C1—S1—N1	118.1 (2)
C8—C9—C10—C11	−0.1 (6)	C6—C1—S1—N1	−65.2 (3)
C9—C10—C11—C12	0.3 (6)		

### Hydrogen-bond geometry (Å, °)

**Table:**

*D*—H···*A*	*D*—H	H···*A*	*D*···*A*	*D*—H···*A*
N1—H1N···O1^i^	0.79 (3)	2.22 (3)	2.981 (4)	163 (4)

Symmetry codes: (i) −*x*+1, −*y*+1, −*z*+2.
